# A pipeline for effectively developing highly polymorphic simple sequence repeats markers based on multi‐sample genomic data

**DOI:** 10.1002/ece3.8705

**Published:** 2022-03-06

**Authors:** Hui Wang, Shenghan Gao, Yu Liu, Pengcheng Wang, Zhengwang Zhang, De Chen

**Affiliations:** ^1^ 47836 MOE Key Laboratory for Biodiversity Science and Ecological Engineering College of Life Sciences Beijing Normal University Beijing China; ^2^ State Key Laboratory of Microbial Resources Institute of Microbiology Chinese Academy of Sciences Beijing China; ^3^ 12534 Jiangsu Key Laboratory for Biodiversity and Biotechnology College of Life Sciences Nanjing Normal University Nanjing China

**Keywords:** microsatellite, molecular marker, next‐generation sequencing, resequencing, short tandem repeats, threatened species

## Abstract

Simple sequence repeats (SSRs) are widely used genetic markers in ecology, evolution, and conservation even in the genomics era, while a general limitation to their application is the difficulty of developing polymorphic SSR markers. Next‐generation sequencing (NGS) offers the opportunity for the rapid development of SSRs; however, previous studies developing SSRs using genomic data from only one individual need redundant experiments to test the polymorphisms of SSRs. In this study, we designed a pipeline for the rapid development of polymorphic SSR markers from multi‐sample genomic data. We used bioinformatic software to genotype multiple individuals using resequencing data, detected highly polymorphic SSRs prior to experimental validation, significantly improved the efficiency and reduced the experimental effort. The pipeline was successfully applied to a globally threatened species, the brown eared‐pheasant (*Crossoptilon mantchuricum*), which showed very low genomic diversity. The 20 newly developed SSR markers were highly polymorphic, the average number of alleles was much higher than the genomic average. We also evaluated the effect of the number of individuals and sequencing depth on the SSR mining results, and we found that 10 individuals and ~10X sequencing data were enough to obtain a sufficient number of polymorphic SSRs, even for species with low genetic diversity. Furthermore, the genome assembly of NGS data from the optimal number of individuals and sequencing depth can be used as an alternative reference genome if a high‐quality genome is not available. Our pipeline provided a paradigm for the application of NGS technology to mining and developing molecular markers for ecological and evolutionary studies.

## Graphical abstract

We designed a pipeline for the rapid development of polymorphic SSR markers from multisample genomic data. The pipeline was successfully applied to a globally threatened species, the browneared‐pheasant.

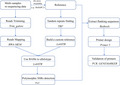

## 1 INTRODUCTION

Simple sequence repeats (SSRs), or microsatellites, are highly variable genetic markers useful for a wide variety of applications in genetic analysis, including genetic mapping, population structure and gene flow analysis, identification of conservation units, and kinship analysis (Gerber et al., 2000; Vashistha et al., 2020; Zamudio & Wieczorek, 2000). Since the first application of SSRs in the 1990s, they have been extensively and continuously used in evolutionary, ecological, and conservation research even in the genomics era (Ali et al., 2019; Allendorf, 2017; Shahabzadeh et al., 2020).

Despite the many advantages of SSR markers, a general limitation to their application is the difficulty of developing polymorphic SSR markers (Squirrell et al., 2003). The development of new SSR markers can basically be divided into the following stages: (1) identification of the sequences containing SSRs; (2) design of PCR primers from flanking regions; and (3) detection of polymorphisms among individuals (Andrés & Bogdanowicz, 2011; Vieira et al., 2016). Traditional approaches, consisting of cloning, cDNA library construction, and Sanger sequencing, are time‐consuming, labor‐intensive, and inefficient in the SSR identification and primer design stages (Zane et al., 2002). Next‐generation sequencing (NGS) can largely overcome these shortcomings, providing effective ways to mine tens of thousands of SSR sequences with sufficient flanking regions. Therefore, NGS technologies are increasingly been applied to obtain novel SSR markers in non‐model organisms (Abdelkrim et al., 2009; Gardner et al., 2011; Wang et al., 2017).

However, previous studies usually used genomic data from only one individual to mine SSR sequences and then randomly selected a few hundred SSRs for polymorphism detection in several individuals through PCR experiments (McCulloch & Stevens, 2011; Zhou et al., 2016). As you can see, it needs to design a lot of primers to manually test their polymorphisms, which is still time consuming and inefficient. The rate of obtaining polymorphic SSR markers is still not high (Taheri et al., 2018), especially for threatened species in which among‐individual genetic differences are subtle. Only a small percentage of randomly selected loci were highly polymorphic and easy to amplify (e.g., Hou et al., 2018; Yang et al., 2017). Thus, the limiting step for SSR development through NGS technologies is no longer SSR identification or primer design, but instead, detection and screening of polymorphic loci. One strategy to break this limitation is to track SSR polymorphisms before PCR experiments, which can be done by sequencing multiple individuals. One idea is to develop primer sequences for every SSR loci from each individual, the primer sequences were then used to identify intersectional SSR loci, these SSR loci were extracted to evaluate their polymorphism (e.g., Cui et al., 2018; Fox et al., 2019). With the development of NGS software, a more straightforward way is to align sequence reads from all individuals to a reference genome to identify polymorphic SSR loci before developing primers (e.g., Guo et al., 2020). This is still a rather new point of view, the effects of using different number of individuals, different sequence depth, and the quality of the reference genome on the yield of polymorphic SSR markers has rarely been explored, especially for threatened species.

The aim of this study is to develop an effective pipeline for the rapid development of highly polymorphic SSR markers from multi‐sample genomic data. We used a subset of the population genomic data from a global threatened galliform bird, the brown eared‐pheasant (*Crossoptilon mantchuricum*), which has very low genomic diversity (Wang et al., 2020). We showed that our pipeline can effectively discover polymorphic SSR markers from such a species and successfully estimate its population structure with the developed SSRs, which showed the same result from genomic data (Wang et al., 2020). Furthermore, we evaluated the effect of different numbers of individuals and sequencing depth on the SSR mining results and assembled a reference genome using multi‐sample low‐depth data instead of single‐sample high‐depth data, which could be a reasonable strategy balancing the sampling and sequencing costs, especially for species without a reference genome.

## 2 MATERIALS AND METHODS

### 2.1 Data and sample collection

For the in silico mining of polymorphic SSRs, we used the assembled genome of one individual and resequencing data (~20 X) of 20 individuals of *C*. *mantchuricum* downloaded from the National Genomics Data Center (BioProject number: PRJCA003284, https://bigd.big.ac.cn/?lang=en), the sample information and accession number of each sample can be seen in Table S1. All the 20 individuals used in this study are not closely related (Wang et al., 2020).

For the experimental validation of the SSR markers, a total of 30 wild individuals of *C*. *mantchuricum* were sampled from Hebei (*n* = 6), Beijing (*n* = 2), Shanxi (*n* = 15), and Shaanxi (*n* = 7) in China. The tissue or blood samples were preserved at −80°C for long‐term storage. Genomic DNA was extracted using a DNA extraction kit (TianGen Biotech, Beijing, China) following the manufacturer's instructions.

### 2.2 In silico mining of polymorphic SSRs

**FIGURE 1:**
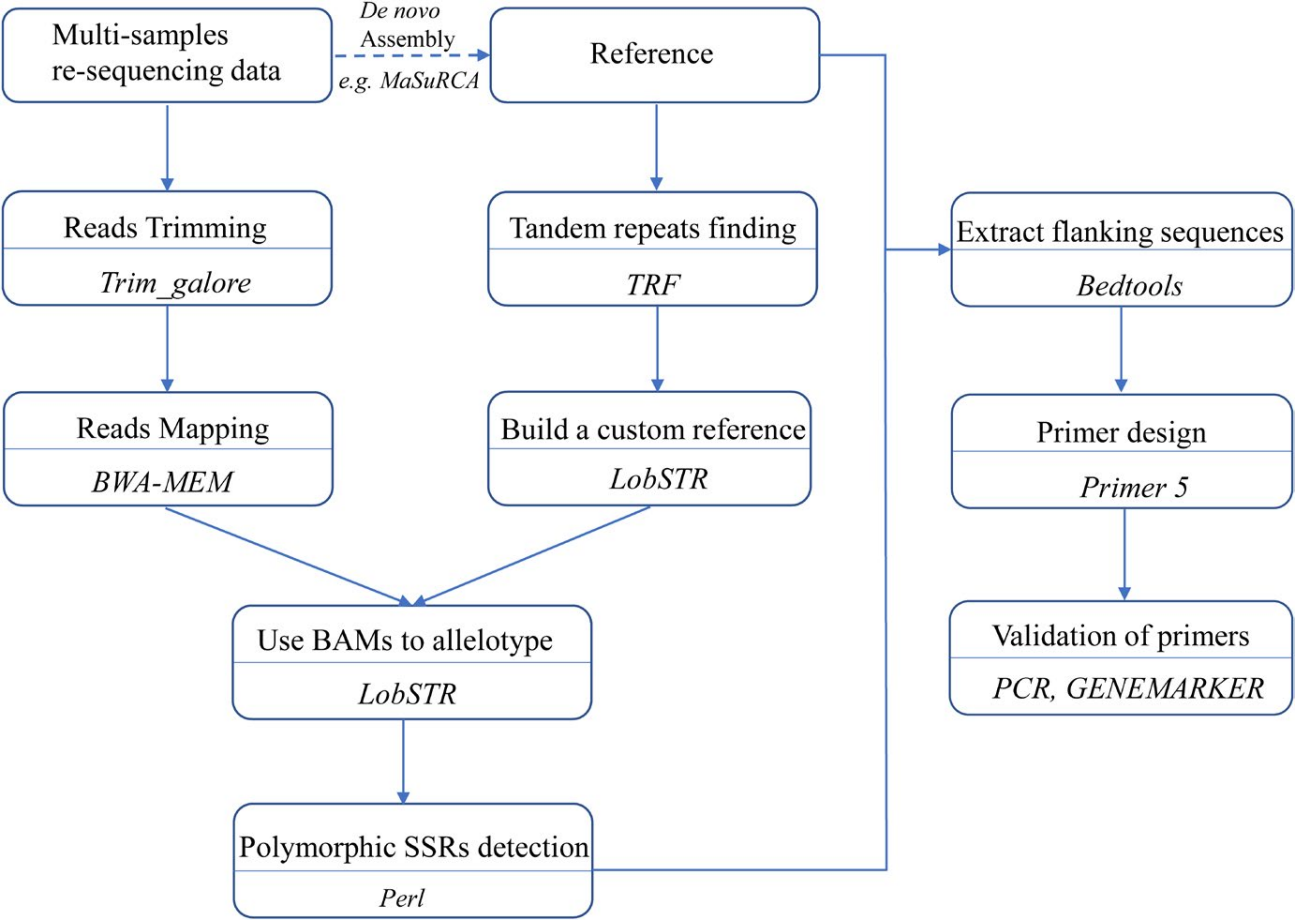
Workflow for in silico microsatellite mining, polymorphism discovery, and primer design using a series of commonly used software programs (shown in italics). The pipeline takes multi‐sample resequencing data in FASTQ format and reference genome in FASTA format as input data (the reference genome can be generated with assembly software such as MaSuRCA from multi‐sample resequencing data for species whose reference genomes were unavailable)

We developed a pipeline using commonly used NGS software to identify polymorphic SSRs from resequencing data of multiple individuals (Figure 1). First, we identified tandem repeats in the reference genome of *C*. *mantchuricum* using Tandem Repeats Finder (TRF) v 4.09 (http://tandem.bu.edu/trf/trf.html) (Benson, 1999) using the following options: alignment score for match, mismatch, indel: 2, 7, 7; PM: 80; PI: 10; minimum alignment score: 50; max period: 500. After tandem repeats identification, we obtained a BED file with our custom set of tandem repeats. Then, we used lobstr_index.py in lobSTR v 4.0.6 (Gymrek et al., 2012) and the BED file to build a custom lobSTR reference for *C*. *mantchuricum* (http://lobstr.teamerlich.org/best‐practices‐custom‐reference.html). Meanwhile, the raw reads of the 20 individuals of *C*. *mantchuricum* were filtered with Trim Galore v 0.5.0 (Krueger, 2012) with default parameters, and clean reads were mapped to the *C*. *mantchuricum* reference genome with BWA‐MEM v 0.7.17‐r1188 (Li & Durbin, 2009) to generate BAM files. Then, we used these BAM files and the custom reference as input for lobSTR (Gymrek et al., 2012) to run allelotypes. After allelotyping, we used a custom Bash script (Appendix S1) to select polymorphic SSR loci from the VCF file generated by lobSTR.

We used a very strict criterion to select SSRs for subsequent experimental validation. First, we focused only on the “perfect” SSRs (uninterrupted run of repeats) (Sharma et al., 2007) that can be successfully genotyped across all individuals (NS = 20) to avoid PCR failure and null alleles. Second, we restricted the motif length to 3–5 bp to avoid genotyping error. Third, we selected SSRs with high polymorphism, that is, the number of alleles for each locus ≥5 (see results), among which 34 potential polymorphic loci comprising different motif lengths were selected for downstream analyses.

### 2.3 SSR primer design and experimental validation

First, we used BEDTools v 2.26.0 (Quinlan & Hall, 2010) to extract 350 bp flanking sequences on both ends of the 34 SSRs from the reference genome. Then, primers were designed in the flanking regions of each SSR locus using Primer Premier v 5 (Lalitha, 2000) with the following parameters: (1) primer lengths ranging from 18 to 27 bp; (2) product sizes ranging from 100 to 500 bp; (3) melting temperature (Tm) ranging from 55°C to 62°C and the differences of Tm between forward and reverse primers <2°C; and (4) GC content ranging from 40 to 60%.

Next, trial polymerase chain reaction (PCR) was conducted in 4 individuals of *C*. *mantchuricum* to test whether the newly designed SSR markers were amplifiable. PCR amplification was performed in a 10‐μl reaction volume containing 0.5 µl of genomic DNA, 5 µl of TianGen Biotech Taq Master Mix, 4 µl of ddH_2_O, 0.25 µl of forward primer (10 µM) and 0.25 µl of reverse primer (10 µM). The PCR amplification programs were as follows: DNA initial denaturation at 95°C for 5 min; 35 cycles of 94°C for 40 s, annealing temperature of specific primer (Table S2) for 30 s, 72°C for 30 s; and a final step at 72°C for 5 min. The PCR products were detected by 2% agarose gel electrophoresis. As a result, we obtained 30 loci that were reliably amplified.

We randomly selected 20 of 30 SSR candidates to synthesize fluorescently labeled forward primers (5′‐FAM, HEX, ROX·; Beijing Genomics Institute, Beijing, China) and performed PCR amplification of all 30 individuals of *C*. *mantchuricum* as described above. The PCR products were sent to Qingke Biotech (Beijing, China) for SSR genotyping detection. Allele scoring for each marker was performed with Genemarker v 2.2.0 (Holland & Parson, 2011).

### 2.4 Statistical and population structure analyses

Genetic parameters such as the number of alleles (*N*_a_), polymorphism information content (PIC), observed heterozygosity (*H*_o_), and expected heterozygosity (*H*_e_) were calculated by Cervus v3.0 (Marshall et al., 1998). The frequency of null alleles was estimated using FreeNA (Chapuis & Estoup, 2007). Linkage equilibrium (LD) were tested using Genepop (Rousset, 2008) with the following parameters: dememorization = 10,000, batches = 20, iterations per batch = 5000. The Bonferroni correction for *p* value was done by Myriads v1.2 (Carvajal‐Rodríguez, 2017).

Population structure analyses were performed using principal coordinate analysis (PCoA) in GenAlEx v6.5 (Peakall & Smouse, 2006) and the model‐based software program STRUCTURE v2.3.4 (Pritchard et al., 2000). The number of subpopulations (K) was set to range from 1 to 10, and for each K, 10 replications were tested. For each run, a burn‐in period was set to 100,000 with 100,000 MCMC iterations. The log probability of the data (LnP(D)) was calculated to confirm the convergence. To determine the most likely value of K, the Evanno method (Evanno et al., 2005) was used via the online program STRUCTURE HARVESTER (http://taylor0.biology.ucla.edu/structureHarvester/) (Earl & Vonholdt, 2012). Genetic differentiation among the populations was calculated with the Weir and Cockerham (1984) estimator of the fixation index (*F*_st_) using FSTAT v2.9.4 and 1,000 permutations were used to test for significant differences (Goudet, 1995; Weir & Cockerham, 1984).

### 2.5 Effects of the number of individuals and sequencing depth on SSR mining

To test the effect of the number of individuals on SSR mining, we randomly selected 2, 4, 6, 8, 10, 12, 14, 16, and 18 individuals from the 20 individuals (sequencing depth ~20X) to perform the same analyses as described above (Figure 1). Second, we fixed the number of individuals as 10 (the optimal number of individuals based on our results) to explore the effect of sequencing depth. The average sequencing depth of each sample was calculated by the tool “bamdst” (https://github.com/shiquan/bamdst). Then, we used SAMtools v1.9 (Li et al., 2009) to randomly generate 2.5X, 5X, 7.5X, 10X, 12.5X, 15X, 17.5X, and 20X resequencing data for each of the 10 individuals and performed the same analyses (Figure 1). For each analysis, we focused only on the SSR loci that existed in all selected individuals and exhibited at least two alleles. To evaluate the SSR mining results, we calculated two parameters: the number of polymorphic SSRs and the Na for each SSR locus. Then, we used R v4.0.2 (R Core Team, 2020) to draw line charts and violin plots to visualize the increasing trend of these two parameters to estimate the optimal values for the number of individuals and sequencing depth. The magnitude of change of the average Na between different individuals and sequencing depth was assessed using Cohen's d effect size analysis. A value of 0.20 is considered a small effect, 0.50 is considered a medium effect (Cohen, 1992).

### 2.6 SSR mining using multi‐sample low‐depth resequencing data without a prior/known reference genome

Generally, a high‐quality reference genome is necessary to map resequencing data and to develop SSR markers (Hou et al., 2018). However, the assembly of an eligible reference genome usually requires deep sequencing >100X from the same individual, which results in considerable additional cost (Desai et al., 2013). To fully utilize the multi‐sample resequencing data and reduce the sequencing cost, we derived the idea used in pan‐genome studies and tried to use multi‐sample low‐depth data to assemble a “consensus” reference genome of *C*. *mantchuricum*. 10X resequencing data of each ten individual (100 X data in total, the optimal number of individuals and sequencing depth based on our results) were used for de novo assembly of the *C*. *mantchuricum* genome with MaSuRCA assembler v3.4.2 (Zimin et al., 2013). MaSuRCA is an overlap‐layout‐consensus (OLC) algorithm‐based assembler that tolerates differences such as SNPs, heterozygotes, and sequencing errors to generate consensus. This feature enables it to generate a consensus assembly by integrating multi‐sample low‐depth data as conventional deep sequencing data. We used the assembled genome as a reference and carried out SSR mining using resequencing data following the designed pipeline (Figure 1). To test the validity of the assembled “consensus” genome on SSR mining results, we calculated the number of polymorphic SSRs and the Na for each SSR locus and compared these SSRs developed with the “consensus” genome to those SSRs developed with the high‐quality genome. In addition, we also compared the ±350 bp flanking sequences on both ends of all the polymorphic SSRs with BLASTN v2.5.0+ (Altschul et al., 1990) to identify the intersection of polymorphic SSRs extracted from the two reference genomes. The SSR loci with >95% identity and >500 bp alignment length were considered the same loci.

## 3 RESULTS

### 3.1 Distribution of SSR types and allele number of *Crossoptilon mantchuricum*

**FIGURE 2:**
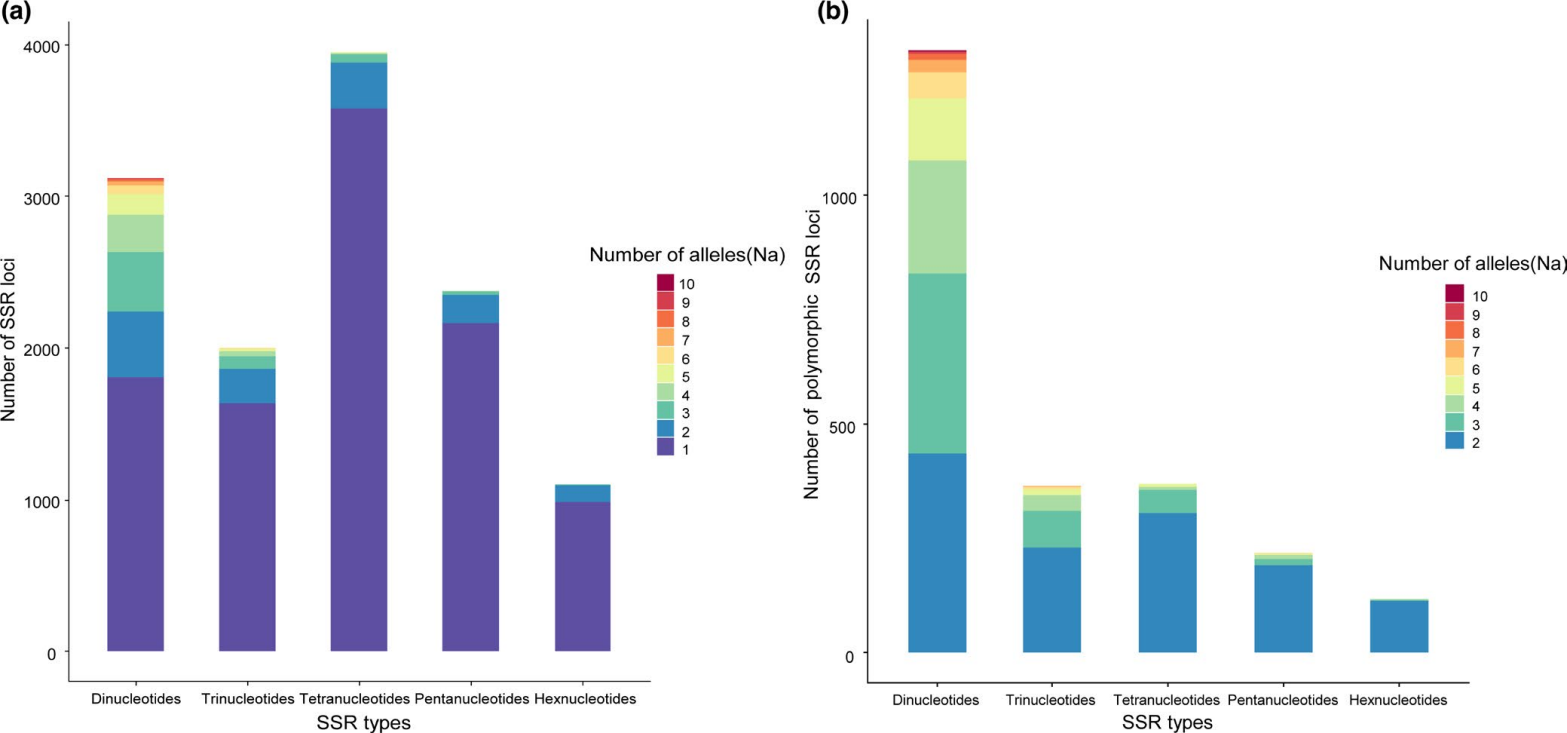
Distributions of SSR types and the number of alleles (Na) of 20 *C*. *mantchuricum* individuals. (a) All SSR loci. (b) Polymorphic SSR loci

Using our designed pipeline, we identified 228,728 tandem sequence repeats in the reference genome of *C*. *mantchuricum*. After genotyping 20 individuals with lobSTR, we found a total of 12,549 “perfect” SSR loci (motif length from 2 to 6 bp) that could be successfully genotyped across all samples. Among these SSRs, the most abundant repeat motifs were tetranucleotides (3947, 31.45%), followed by dinucleotides (3120, 24.86%), pentanucleotides (2380, 18.97%), trinucleotides (2000, 15.94%), and hexanucleotides (1102, 8.78%; Figure 2, Table S3).

The average number of alleles (*N*_a_) for the 12,549 SSRs was only 1.36, and 81.01% of the loci were monomorphic (Figure 2). The proportion of polymorphic loci for dinucleotide repeats was higher than that for other types (Figure 2). However, dinucleotide microsatellites are easily subjected to mistyping due to polymerase slippage during PCR (Schlötterer & Tautz, 1992). To develop SSR markers with strong stability and a low genotyping error rate, we only focused on SSRs with motif lengths ranging from 3 to 5 bp in this exploratory study. We obtained 952, 228, 83, 34, and 10 loci when we restricted the minimum Na to 2, 3, 4, 5, and 6, respectively (Table S3). Based on the above results, we selected the 34 SSRs with an *N*_a_ ≥ 5 to perform downstream analyses.

The 34 candidate polymorphic SSR loci consisted of 22 trinucleotide, seven tetranucleotide, and five pentanucleotide repeats, among which 30 loci were successfully amplified with designed primers (88.24%). We randomly selected 20 loci for polymorphism detection. Twenty SSR loci consisted of 14 trinucleotide, four tetranucleotide, and two pentanucleotide repeats (Table S2).

### 3.2 Descriptive statistical and population structure

The PIC values of the 20 SSR loci ranged from 0.315 to 0.707 with an average of 0.532. The *N*_a_ ranged from 2 to 6 with an average of 4.2, the *H*_o_ ranged from 0 to 0.633 with an average of 0.345, and the He ranged from 0.398 to 0.760 with an average of 0.597 (Table 1).

**TABLE 1:** Summary of the observed allele number (*N*_a_), sample size (*N*), observed and expected heterozygosity (*H*o and *H*_e_), and polymorphism information content (PIC) for 30 individuals of brown eared‐pheasants

No.	Marker Name	*N*_a_	*N*	*H*_o_	*H*_e_	PIC
1	CM1	3	30	0.300	0.605	0.528
2	CM2	5	30	0.567	0.726	0.667
3	CM3	3	30	0.033	0.406	0.332
4	CM7	4	30	0.167	0.547	0.475
5	CM8	6	30	0.400	0.714	0.658
6	CM9	3	30	0.400	0.453	0.381
7	CM10	5	30	0.567	0.692	0.624
8	CM11	4	30	0.233	0.551	0.481
9	CM12	3	30	0.267	0.473	0.420
10	CM14	5	30	0.567	0.744	0.684
11	CM15	3	30	0.167	0.581	0.508
12	CM16	5	30	0.567	0.724	0.663
13	CM19	2	30	0.000	0.398	0.315
14	CM20	4	29	0.103	0.470	0.423
15	CM25	4	30	0.400	0.481	0.437
16	CM26	4	30	0.300	0.584	0.513
17	CM27	6	29	0.276	0.629	0.562
18	CM30	6	30	0.633	0.733	0.675
19	CM32	5	30	0.533	0.760	0.707
20	CM33	4	28	0.429	0.660	0.581
Mean		4.2	29.8	0.345	0.597	0.532

**FIGURE 3:**
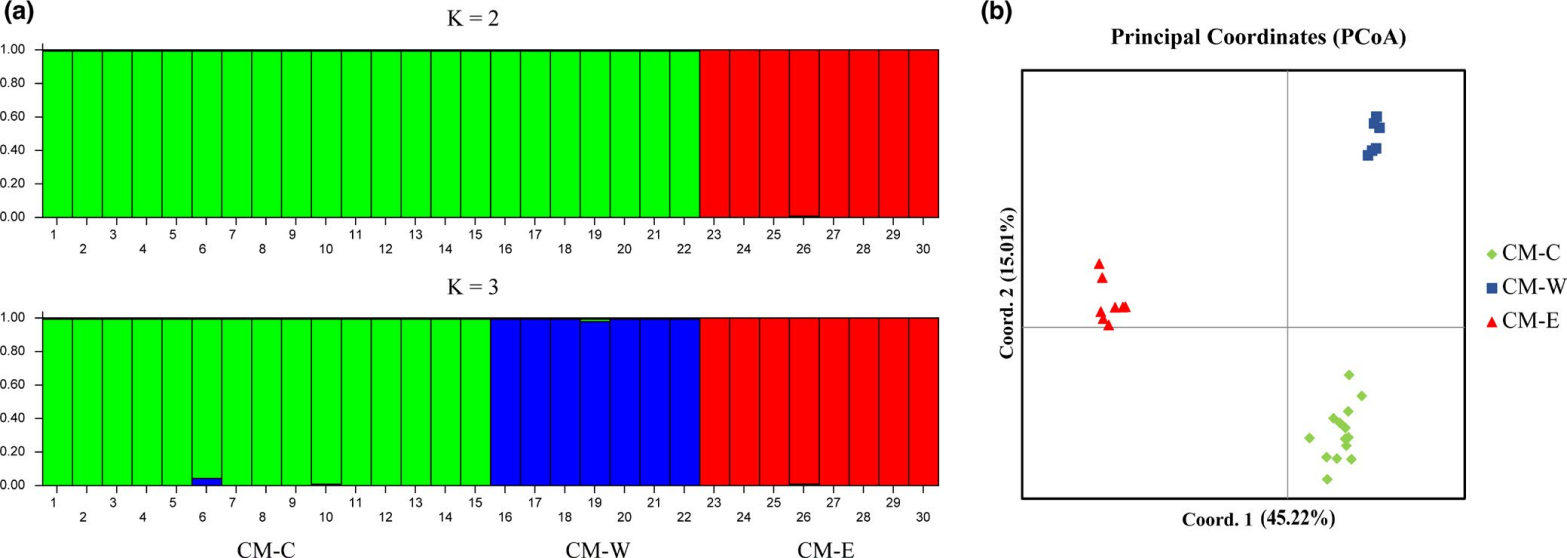
Population structure and principal coordinate analysis (PCoA) of 30 brown eared‐pheasants based on 20 SSR markers. (a) Population structure of *K* = 2 and *K* = 3 inferred by Bayesian clustering approaches. Samples of 30 brown eared‐pheasants were from Shanxi (*n* = 15; 1–15), Shaanxi (*n* = 7; 16–22), Hebei and Beijing (Hebei: *n* = 6, Beijing: *n* = 2; 23–30). (b) Principal coordinate analysis (PCoA) of 30 brown eared‐ pheasants. CM‐C: Shanxi (*n* = 15; green); CM‐W: Shaanxi (*n* = 7; blue); CM‐E: Hebei and Beijing (Hebei: *n* = 6, Beijing: *n* = 2; red)

PCoA divided the 30 brown eared‐pheasants into three genetic clusters (Figure 3). The first two principal coordinates (PCos) explained 60.23% of the total variance (45.22% and 15.01%, respectively). In our 10 independent structure analyses to estimate *K*, the values of LnP(D) increased sharply from *K* = 1 to *K* = 3, and delta K analysis revealed a peak at *K* = 2 (Figure S1), suggesting at least two clusters. When *K* = 3, the three populations of brown eared‐pheasant can also be separated very well (Figure 3), which is in accordance with the PCoA results. We identified the three brown eared‐pheasant populations as CM‐W (Shaanxi, *n* = 7), CM‐C (Shanxi, *n* = 15), and CM‐E (Hebei and Beijing, *n* = 8), which represented the western, central, and eastern populations, respectively. The pairwise *F*_st_ values among the three populations ranged from 0.364 to 0.742 and all the pairwise *F*_st_ values were significantly greater than zero (Table S4).

The population structure analyses showed high genetic differentiation among the three populations of the brown eared‐pheasant, so we estimated null allele frequency of the 20 loci in three population separately. The prevalence of null alleles for most loci is low (<0.05), except for loci CM27 and CM12 in the central and eastern populations, respectively (>0.2, Table S5). The average null allele frequency of the 20 loci is low (<0.05) among three populations. Such a low frequency of null alleles only has slight impact on population genetic analyses (Carlsson, 2008; Chapuis & Estoup, 2007; Dakin & Avise, 2004). Only 2 out of 570 tests for LD were significant after Bonferroni correction (CM8–CM14 and CM11–CM25 in the central population). These four SSR loci located on different scaffolds, the observed LD might be caused by the low genomic diversity (Wang et al., 2020) and/or small sample size rather than true linkage.

### 3.3 Effect of the number of individuals and sequencing depth on mining SSRs

**FIGURE 4:**
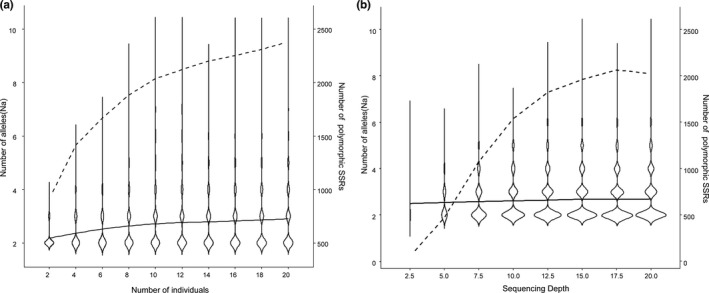
The increasing trends of the number of alleles (solid line) and the number of polymorphic SSRs (dotted line) depending on the number of individuals (a) and sequencing depth (b)

The number of individuals had a great influence on the calculated *N*_a_, while the sequencing depth had a great influence on the obtained number of polymorphic SSRs (Figure 4). When the number of individuals reached 10, the increasing trend of Na slows down (Cohen's d: Na_2_ vs. Na_10_: −0.61 (medium), Na_10_ vs. Na_20_: −0.12 (small)), and the number of polymorphic SSRs reached approximately 2,037 (85.48% compared to using 20 individuals; Figure 4a). Thus, we fixed the number of individuals to ten to explore the effect of sequencing depth. Our results showed that Na remained nearly stable with increasing depth (2.460–2.695; Cohen's d: Na_2.5x_ vs. Na_20x_: −0.23 (small)), while the number of polymorphic SSR loci increased rapidly (from 50 to 2022, Figure 4b, Table S6). The increase in the number of SSR loci slowed when the sequencing depth reached 10–12.5 X (Figure 4b), when sufficient (1539–1825) polymorphic SSR loci were identified.

### 3.4 SSR mining results using multi‐sample low‐depth resequencing data without a prior/known reference genome

The draft “consensus” genome assembly based on resequencing data of ten individuals (~10X for each individual) comprised 73,187 scaffolds with a total length of 1.06 Gb. The genome sequence had 41.8% GC content. The scaffold and contig N50 were 132.68 kb and 100.00 kb, respectively. Using this assembled genome as the reference genome, we found a total of 9306 “perfect” SSR loci, of which 1590 (17.09%) were polymorphic. In comparison, the number of polymorphic SSR loci extracted from the canonical reference genome was 1,539, which was similar to the assembled genome (1590 vs. 1539, Figure S2). The BLASTN analysis showed that nearly 80% (1216/1539) of these loci overlapped. The average Na of all the SSR loci from the assembled genome was 1.275, which showed no significant difference compared to that of the SSR loci obtained using the canonical reference genome (average *N*_a_ = 1.271; *t*‐test: *T* = 0.37, *df* = 18460, *p* = .71).

## 4 DISCUSSION

Although genome‐wide SNPs have become more and more popular for studies of population genetics, SSRs are still valuable genetic markers due to their high polymorphism, low DNA template demands, relatively easy application, along with well‐developed and simple statistical analyses (Hodel et al., 2016; Zane et al., 2002). There are several scenarios where SSRs are comparable with genome‐wide SNPs. For example, studies require parentage and kinship determination in behavioral ecology and genetic management do not require high marker density, but benefit more from large number of samples (de Deus et al., 2021). It is impractical and expensive to genotype thousands of individuals using genome‐wide SNPs and it is hard to update the dataset if small numbers of new individuals are added. Conversely, once the SSR markers has been developed, it would be much easier and more economical to genotype additional individuals (Puckett, 2017). In addition, SSR is still the most widely used genetic marker in forensic identifications and noninvasive genetic studies of endangered species from degraded samples owing to its low quantity/quality DNA template demand and high reproducibility results (Lampa et al., 2013; Willows‐Munro & Kleinhans, 2020). We can acquire sufficient DNA for SSR genotyping even in degraded samples such as eggshells, feathers, and feces (Baus et al., 2019). Furthermore, a strong background in computing skills and bioinformatics is needed to deal with the large quantity of SNPs, whereas researchers can complete SSR analyses with limited computing skills on a laptop computer (Hodel et al., 2016). For all these reasons, microsatellites remain a good choice for many systems and questions and they will continue to be used extensively in ecology, evolution, and conservation in the future.

In this study, we developed a pipeline to mine polymorphic SSR markers based on NGS data from multiple individuals of the target species. The pipeline was successfully applied to a globally threatened species with very low genomic diversity (Wang et al., 2020). We further evaluated the effect of different numbers of individuals and sequence depths on the SSR mining results to suggest a reasonable strategy balancing data generation and cost. Additionally, we showed that the pipeline worked well even without a high‐quality reference genome, which further extended its application range and decreased the cost of developing applicable polymorphic SSR markers.

We found that the average Na was only 1.36 for the brown eared‐pheasant at the genome scale, and less than 10% of SSRs had more than two alleles among 20 individuals (Figure 2, Table S3). Therefore, it will be rather inefficient to filter polymorphic SSR markers through experimental validation from randomly chosen SSR loci, which is the commonly used SSR marker development method using NGS data (Table 2) (Hou et al., 2018; Huang et al., 2015; Taheri et al., 2018). For example, Zhu (2014) used blood transcriptome from one male brown eared‐pheasant to develop SSR markers, he randomly selected 118 SSR loci to design primers, only 5% (6/118) are polymorphic (Table 2), the average Na was 2.17 among 24 individuals. Our pipeline took advantage of resequencing data from multiple individuals and detected highly polymorphic SSRs among these individuals prior to experimental validation, significantly improved the efficiency and reduced experimental effort in developing polymorphic SSR markers (Table 2). Except for the four SSR loci that failed to pass our trial PCR (which could be improved if we redesigned primers), the other 30 randomly selected SSR markers were very stable during PCR experiments. Our following test showed that all of the 20 randomly selected SSR markers are polymorphic, the Na ranged from 2 to 6, with an average of 4.2 among 30 individuals, which was significantly higher than the average Na on the genome scale.

**TABLE 2:** A comparison of different SSR marker develop methods, including species, SSR marker develop methods (Tra‐NGS: Traditional NGS method based on one individual), number of PCR primers tested (Pri), number of amplifiable PCR primers (Amp), percentage of primers which were amplifiable (Amp/Pri), number of primers selected to test polymorphism (Amp‐sel), number of polymorphic primers (Pol), percentage of amplifiable primers which were polymorphic (Pol/Amp‐sel), percentage of primers which were amplifiable and polymorphic (Suc), literature reference (Ref)

Species	Method	Pri	Amp	Amp/Pri	Amp‐sel	Pol	Pol/Amp‐sel	Suc	Ref
*Crossoptilon mantchuricum*	Tra‐NGS	118	118	100%	118	6	5%	5%	Zhu (2014)
*Liocichla omeiensis*	Tra‐NGS	600	99	17%	52	24	46%	8%	Yang et al. (2017)
*Dromaius novaehollandiae*	Tra‐NGS	144	143	99%	143	49	34%	34%	Koshiishi et al. (2021)
*Crossoptilon mantchuricum*	This study	34	30	88%	20	20	100%	88%	This study

Our results showed that the increase trend of Na slows down after subsampling more than 10 individuals (Figure 4a). Given the extremely low genetic diversity of the brown eared‐pheasant, fewer individuals should be sufficient for other species. For example, we have already developed highly polymorphic SSR markers for the Daurian redstart (*Phoenicurus auroreus*) and the Chinese penduline tit (*Remiz consobrinus*) following the pipeline, both using resequencing data of eight individuals (in preparation). Although a higher sequencing depth can increase the number of polymorphic SSR loci, our results demonstrated that a low to medium depth (10X–12.5 X) can generate large numbers of highly polymorphic loci from such species with low genomic diversity (Figure 4b). For other species with larger population size, a 10X sequencing depth may be enough to obtain a sufficient number of polymorphic SSR loci.

Despite the rapid development of sequencing technology, there are still many species for which reference genomes are unavailable. Assembling a high‐quality reference genome is usually a demanding project requiring deep sequencing depth of a single individual (>100X). Since the total length of SSR markers is generally short, the development of SSRs does not require a high‐quality reference genome. Previous studies usually used sequence reads from the sequenced individual to assembly a draft genome to mine SSR sequences (Koshiishi et al., 2021; Yang et al., 2017). As for the multi‐sample strategy, we used multi‐sample low‐depth data to generate a draft reference genome inspired by pan‐genome strategies, The scaffold and contig N50 of the assembled genome were approximately 134 kb and 100 kb, respectively, which are lower than those of the canonical high‐quality genome (scaffold/contig N50: 3,632.75 kb/112.76 kb; Wang et al., 2020). Although the quality of the assembled genome was lower than that of the canonical high‐quality genome, the numbers of polymorphic SSR loci mined with our pipeline were very similar, and approximately 80% of SSR loci overlapped, which might be higher if we lower the length standard of the flanking sequence. Furthermore, the average Na between SSR markers from the assembled genome and SSR markers from the canonical high‐quality reference genome showed no significant difference, which means that the distribution of SSRs in the “consensus” genome was highly consistent with the high‐quality reference genome. Overall, the use of a reference genome by using a “consensus” genome strategy from multi‐sample low‐depth data can yield approximately the same number of polymorphic SSR loci, which can further reduce the cost of developing SSR markers. As the sequencing cost of NGS has dramatically declined since its invention (https://www.genome.gov/about‐genomics/fact‐sheets/DNA‐Sequencing‐Costs‐Data), the cost for resequencing 10X data from 10 individual of birds is about $1100. If we randomly design 100 primers to detect polymorphism on 4 individuals (many studies used this strategy), the cost is about $550 for primer synthesis, and $1000 for sanger sequencing, which are similar or even slightly higher than the resequencing cost.

The brown eared‐pheasant is a globally threatened species distributed in China (Zheng, 2015), and polymorphic SSR markers for this species are still unavailable. In this study, we developed 20 new SSR markers. The PIC indicated that 12 markers were highly informative (PIC > 0.5), and the other eight were reasonably informative (0.5 < PIC < 0.25) (Botstein et al., 1980). These SSR loci were successfully applied to the population structure analysis for the brown eared‐pheasant. The PCoA and structure analysis revealed three populations across the range of the brown eared‐pheasant (Figure 3), in accordance with the results from genomic SNP data (Wang et al., 2020). However, the structure analysis revealed a peak of delta *K* at *K* = 2, while it separated the three populations very well when *K* = 3 (Figure 3). Previous studies found that there was a strong bias toward selecting *K* = 2 using the delta *K* method (Cullingham et al., 2020). In addition, uneven sample sizes between subpopulations may lead to the underestimation of delta *K* (Puechmaille, 2016). Our Fst estimations also showed high genetic differentiation among the three populations (Table. S4), which indicated that the brown eared‐pheasant should be divided into three genetic populations. Furthermore, our newly developed SSR markers can be used in various aspects of conservation genetics, such as genetic background analysis and genealogy establishment of captive brown eared‐pheasants and individual identification in wild brown eared‐pheasant populations. In addition, we focused only on SSRs with motif lengths ranging from 3 to 5 bp in this exploratory research. Higher polymorphic dinucleotide SSRs can be easily obtained from our pipeline for further research.

## 5 CONCLUSION

In this study, we developed a pipeline for the rapid development of polymorphic SSR markers using multi‐sample genomic data. Our pipeline can be easily applied in non‐model species in which genomic information is unknown and in threatened species in which genetic diversity is extremely low. Our pipeline provided a paradigm for the application of NGS technology in mining molecular markers for ecological and evolutionary studies.

## CONFLICT OF INTEREST

None declared.

## AUTHOR CONTRIBUTIONS

**Hui Wang:** Formal analysis (equal); Software (equal); Writing – original draft (lead); Writing – review & editing (equal). **Shenghan Gao:** Resources (supporting); Software (equal); Writing – review & editing (supporting). **Yu Liu:** Software (equal); Writing – review & editing (equal). **Pengcheng Wang:** Resources (equal); Writing – review & editing (supporting). **Zhengwang Zhang:** Funding acquisition (lead); Project administration (lead); Writing – review & editing (supporting). **De Chen:** Funding acquisition (lead); Methodology (lead); Project administration (lead); Resources (equal); Software (equal); Writing – original draft (supporting); Writing – review & editing (equal).

## Supporting information

Fig S1

Fig S2

Supplementary Material

Table S1‐S6

## Data Availability

The VCF file, the sequences file of the 34 SSR loci used to design primers and the allele scoring results of the 20 SSR loci were deposited in Mendeley, https://doi.org/10.17632/jdkpgspwvt.1.
