# Supplementary figures and images for: A pipeline for effectively developing highly polymorphic simple sequence repeats markers based on multi‐sample genomic data
Source: Ecol Evol. 2022 Mar 6;12(3):e8705. doi: 10.1002/ece3.8705 (PMC8928897; doi:10.1002/ece3.8705)

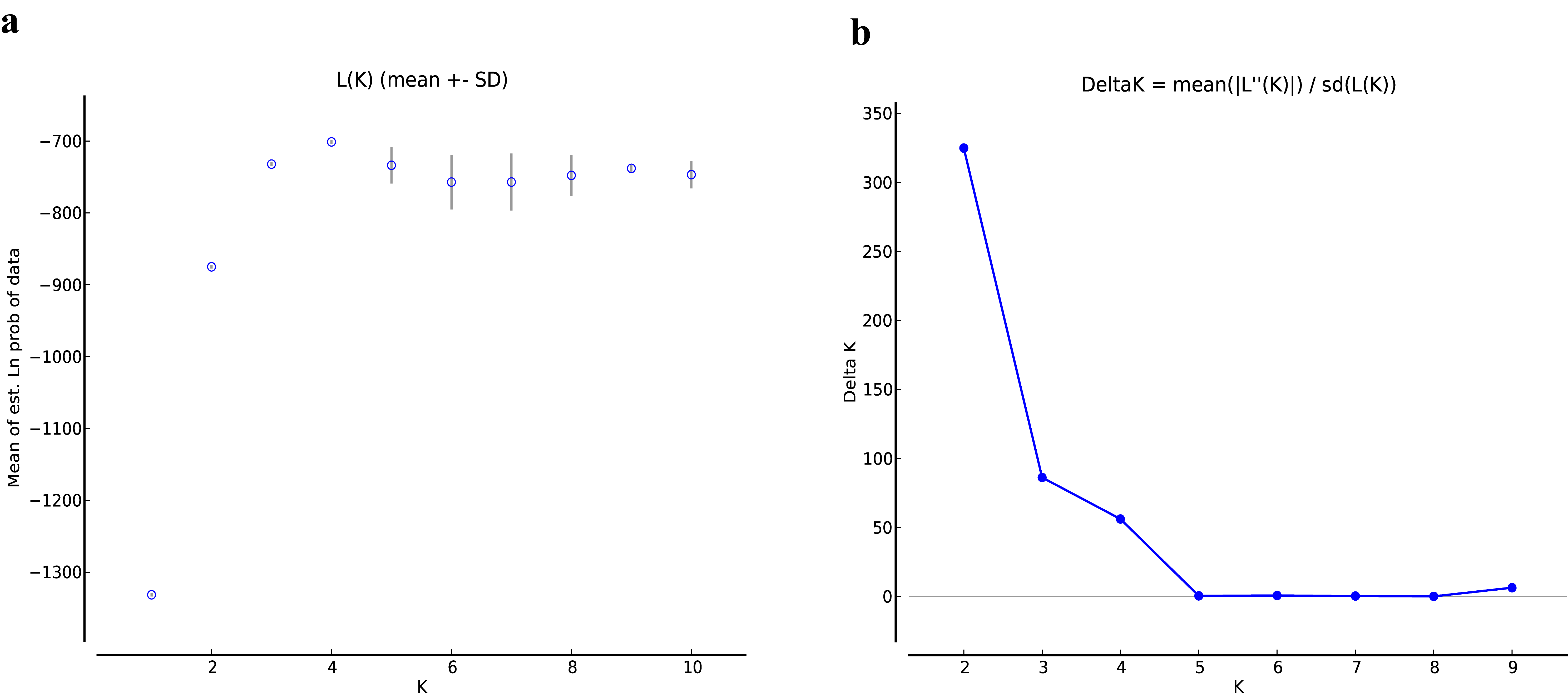

Supplement: Supplementary file 1 — Fig S1 [file ECE3-12-e8705-s002.tif]

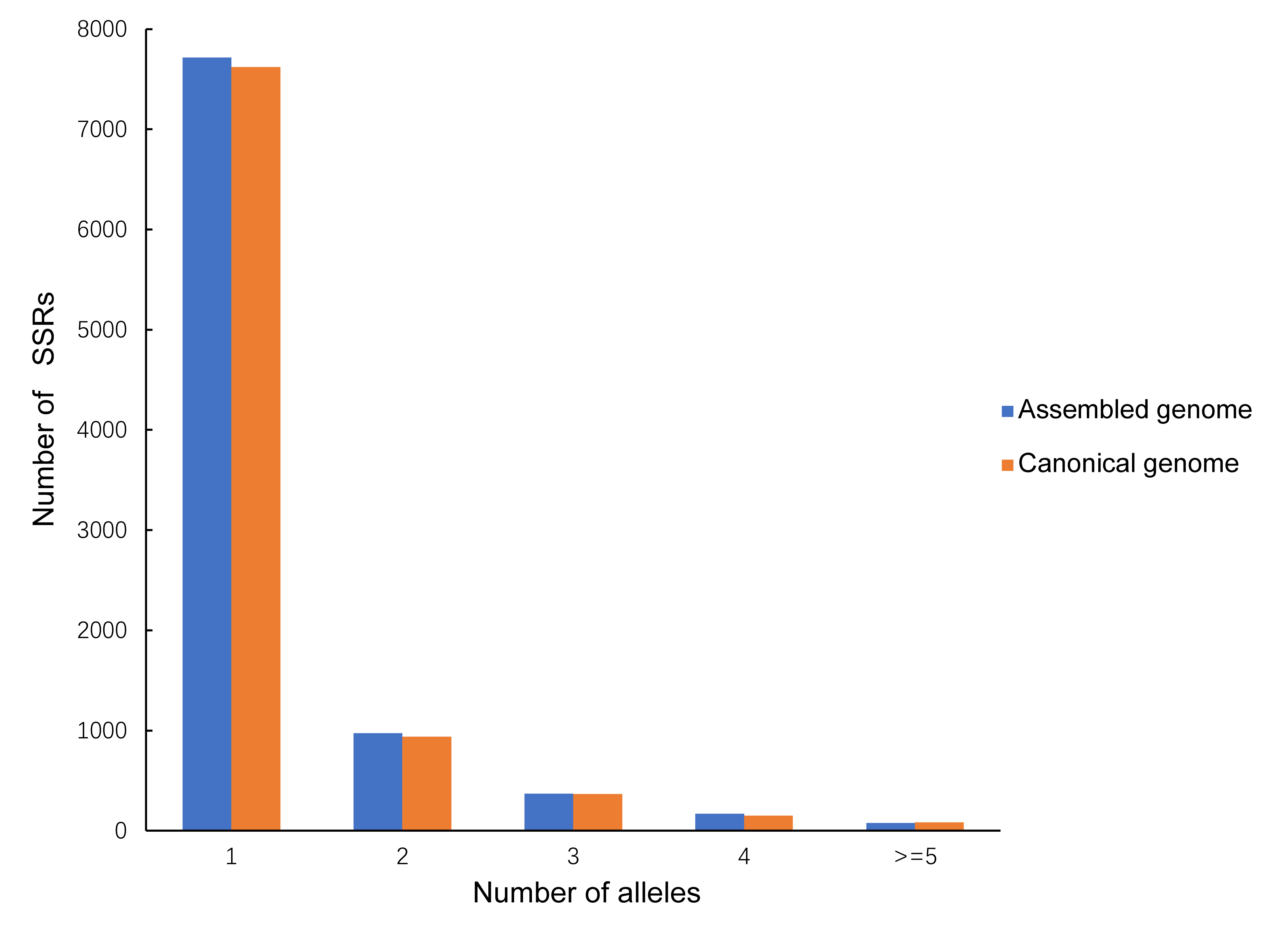

Supplement: Supplementary file 2 — Fig S2 [file ECE3-12-e8705-s003.tif]
