# Supplementary material for: A pipeline for effectively developing highly polymorphic simple sequence repeats markers based on multi‐sample genomic data
Source: Ecol Evol. 2022 Mar 6;12(3):e8705. doi: 10.1002/ece3.8705 (PMC8928897; doi:10.1002/ece3.8705)
Supplement: Supplementary file 3 — Supplementary Material [file ECE3-12-e8705-s001.docx]

#!/bin/bash

##Bash script used to select polymorphic SSR loci from the VCF file generated by lobSTR

##inp => input file oup => output file

##usage : sh get_str.sh /path/to/input_file /path/to/output_file

inp=$1

oup=$2

perl -ne 'if (/MOTIF=[^;]{3,5};/ && /NS=20;/ && /RPA=([\d,]+)/ ){ $rpa=$1; @rpa=split/,/,$rpa; print if (@rpa>=5);}' $inp > $oup.motif3_5.rpa_5
