# Supplementary material for: A pipeline for effectively developing highly polymorphic simple sequence repeats markers based on multi‐sample genomic data
Source: Ecol Evol. 2022 Mar 6;12(3):e8705. doi: 10.1002/ece3.8705 (PMC8928897; doi:10.1002/ece3.8705)
Supplement: Supplementary file 4 — Table S1‐S6 [file ECE3-12-e8705-s004.docx]

Table S1. The sample information and accession number of *C. mantchuricum* downloaded from the National Genomics Data Center.

| Sample index | Location | | Sample ID | Sequencing type | Sequencing Depth | Accession number |
| --- | --- | --- | --- | --- | --- | --- |
| 1 | | Shanxi (Central population) | CMA00 | *De novo* sequencing | 201.75 | SAMC226739 |
| 2 | | Shanxi (Central population) | CMA09 | Re-sequencing | 19.23 | SAMC226719 |
| 3 | | Shanxi (Central population) | CMA11 | Re-sequencing | 19.53 | SAMC226721 |
| 4 | | Shanxi (Central population) | CMA12 | Re-sequencing | 18.26 | SAMC226722 |
| 5 | | Shanxi (Central population) | CMA13 | Re-sequencing | 21.46 | SAMC226723 |
| 6 | | Shanxi (Central population) | CMA14 | Re-sequencing | 19.13 | SAMC226724 |
| 7 | | Shanxi (Central population) | CMA16 | Re-sequencing | 16.03 | SAMC226726 |
| 8 | | Shanxi (Central population) | CMA18 | Re-sequencing | 15.32 | SAMC226727 |
| 9 | | Shaanxi (Western population) | CMAA01 | Re-sequencing | 15.60 | SAMC226700 |
| 10 | | Shaanxi (Western population) | CMAA02 | Re-sequencing | 15.89 | SAMC226701 |
| 11 | | Shaanxi (Western population) | CMAA03 | Re-sequencing | 15.31 | SAMC226702 |
| 12 | | Shaanxi (Western population) | CMAA05 | Re-sequencing | 18.86 | SAMC226704 |
| 13 | | Shaanxi (Western population) | CMAA06 | Re-sequencing | 18.56 | SAMC226705 |
| 14 | | Shaanxi (Western population) | CMAA08 | Re-sequencing | 20.81 | SAMC226707 |
| 15 | | Shaanxi (Western population) | CMAA13 | Re-sequencing | 18.19 | SAMC226712 |
| 16 | | Shaanxi (Western population) | CMAA14 | Re-sequencing | 18.36 | SAMC226713 |
| 17 | | Hebei (Eastern population) | CMH01 | Re-sequencing | 17.40 | SAMC226729 |
| 18 | | Hebei (Eastern population) | CMH05 | Re-sequencing | 18.30 | SAMC226733 |
| 19 | | Hebei (Eastern population) | CMH06 | Re-sequencing | 19.69 | SAMC226734 |
| 20 | | Hebei (Eastern population) | CMH08 | Re-sequencing | 15.51 | SAMC226736 |
| 21 | | Hebei (Eastern population) | CMH09 | Re-sequencing | 18.92 | SAMC226737 |

Table S2. List of sequences of the 20 SSR primer pairs used in the validation of SSR markers.

| No. | Marker name | Primer sequences F/R (5′-3′) | Motifs | Tm (℃) | Product range (bp) |
| --- | --- | --- | --- | --- | --- |
| 1 | CM1 | F: AGACTGCTTTTCCCTACCCAT  R: GCTCCTCATAACAGACTTCCATAA | ATT | 60 | 321-333 |
| 2 | CM2 | F: ACCTTGACATAATGCCCTGA  R: AGAAATCCAACCTGATAGCATA | ATT | 56 | 233-257 |
| 3 | CM3 | F: ATTGCTGTGGCTGCTGACTC  R: GCTTTCAAATGTAGATATTACTTCCA | AAT | 60 | 363-384 |
| 4 | CM7 | F: GTGTTACAGGACATAGGGGAAAG  R: CCCAAGTGACCACGTTGAA | AAT | 60 | 329-347 |
| 5 | CM8 | F: CACATTCTGCCATTCTTCG  R: GACTGTCTCACTGCTAACCAAAC | AAT | 60 | 429-450 |
| 6 | CM9 | F: GTTGAATGTTTGGTCACTGGT  R: CGTCTGGGACTGGGCTA | ATT | 60 | 355-364 |
| 7 | CM10 | F: TAGGGTGGGAGAACAGACAGA  R: CGTTATCCATTGGATGGTTTAGT | AAT | 60 | 364-379 |
| 8 | CM11 | F: CTCTGAGGAGATGGGTTGGT  R: TGATGGAAGGAGGGTGGAT | AAT | 60 | 278-287 |
| 9 | CM12 | F: CCTACTGCCAATGGATGTTC  R: CCAATACCCCAGCACTTTT | TTA | 56 | 374-386 |
| 10 | CM14 | F: TCGCTGTAATATGTTTTGGG  R: AAGCTGGAAGAATAACTGGTCA | ATT | 56 | 186-198 |
| 11 | CM15 | F: AAGACTGAAGACGCTGGTAGTG  R: CGTAGGGTGCAAACAAGAAG | TTA | 60 | 265-277 |
| 12 | CM16 | F: CTGCCAAAAGCATCAAATC  R: TTCATCATCCTAAGCCATCT | TTA | 56 | 326-341 |
| 13 | CM19 | F: AGTGGGGATGCCTTCTGCT  R: TTGAAAAGCAAGGAGGTACTGATA | TAT | 60 | 370-385 |
| 14 | CM20 | F: TCTGTGCTTTGCCTTGAATTT  R: TCAGTGGTGAAGTTTGCTGCT | AAT | 60 | 453-468 |
| 15 | CM25 | F: GCGGTTGTCACATTATCGTTC  R: TGGCTATTAGTGCTGGTCTGG | AAAT | 60 | 392-408 |
| 16 | CM26 | F: TACCATCACCCCTCTGGACAT  R: ATGAATGGACGGACGAGTTG | TCCA | 60 | 126-158 |
| 17 | CM27 | F: CAACTTTGAAGATGCTGAGGAA  R: ATTGGAATGGCTTAGGAACAC | TCTT | 56 | 448-468 |
| 18 | CM30 | F: TAGCTGGTCATTGGTTGGG  R: TCAAGATGCCTGCCTTAGAG | GAAA | 60 | 434-454 |
| 19 | CM32 | F: CTAGACCAGTGCAGTATTAAATGAG  R: AGAGGTTTGGACTGGACATTAG | GAATA | 60 | 335-365 |
| 20 | CM33 | F: CTAACATCTAACCCAAACCTCC  R: CCAGACCCATTGCCACTAT | TCCAT | 60 | 420-435 |

Table S3. Distributions of SSR types and allele numbers of *C. mantchuricum*.

| motif length | No. of allele | No. of loci | Percentage |
| --- | --- | --- | --- |
| 2 | 1 | 1806 | 57.88 |
|  | 2 | 434 | 13.91 |
|  | 3 | 393 | 12.60 |
|  | 4 | 248 | 7.95 |
|  | >=5 | 239 | 7.66 |
|  | sum | 3120 | / |
| 3 | 1 | 1635 | 81.75 |
|  | 2 | 229 | 11.45 |
|  | 3 | 81 | 4.05 |
|  | 4 | 33 | 1.65 |
|  | >=5 | 22 | 1.10 |
|  | sum | 2000 | / |
| 4 | 1 | 3578 | 90.65 |
|  | 2 | 305 | 7.73 |
|  | 3 | 49 | 1.24 |
|  | 4 | 8 | 0.20 |
|  | >=5 | 7 | 0.18 |
|  | sum | 3947 | / |
| 5 | 1 | 2162 | 90.84 |
|  | 2 | 190 | 7.98 |
|  | 3 | 15 | 0.63 |
|  | 4 | 8 | 0.34 |
|  | >=5 | 5 | 0.21 |
|  | sum | 2380 | / |
| 6 | 1 | 985 | 89.38 |
|  | 2 | 112 | 10.16 |
|  | 3 | 4 | 0.36 |
|  | 4 | 1 | 0.09 |
|  | >=5 | 0 | 0.00 |
|  | sum | 1102 | / |

Table S4. Pairwise Fst and *p* values among the three populations of the brown eared-pheasant.

|  | CM-C | CM-W | CM-E |
| --- | --- | --- | --- |
| CM-C | - | 0.001 * | 0.001 * |
| CM-W | 0.364 | - | 0.002 * |
| CM-E | 0.556 | 0.742 | - |

Pairwise Fst values are below diagonal, *p* values above diagonal (**p* < 0.05).

Table S5. Null allele frequency of the 20 loci in three populations of the brown eared-pheasant.

| No. | Marker Name | null allele frequency | | |
| --- | --- | --- | --- | --- |
|  |  | CM-C(n=15) | CM-W(n=7) | CM-E(n=8) |
| 1 | CM1 | 0.0000 | 0.0010 | 0.0000 |
| 2 | CM2 | 0.0000 | 0.0010 | 0.0222 |
| 3 | CM3 | 0.0010 | 0.0010 | 0.0000 |
| 4 | CM7 | 0.0335 | 0.0010 | 0.0010 |
| 5 | CM8 | 0.0224 | 0.0010 | 0.0000 |
| 6 | CM9 | 0.0000 | 0.0000 | 0.1526 |
| 7 | CM10 | 0.0000 | 0.0010 | 0.0000 |
| 8 | CM11 | 0.0000 | 0.0010 | 0.0010 |
| 9 | CM12 | 0.0000 | 0.0000 | 0.2471 |
| 10 | CM14 | 0.0000 | 0.0000 | 0.0000 |
| 11 | CM15 | 0.0010 | 0.0010 | 0.0000 |
| 12 | CM16 | 0.0000 | 0.0000 | 0.0010 |
| 13 | CM19 | 0.0010 | 0.0010 | 0.0010 |
| 14 | CM20 | 0.0010 | 0.0000 | 0.1735 |
| 15 | CM25 | 0.0000 | 0.0010 | 0.0000 |
| 16 | CM26 | 0.0000 | 0.0010 | 0.0010 |
| 17 | CM27 | 0.2196 | 0.0000 | 0.0000 |
| 18 | CM30 | 0.0000 | 0.0000 | 0.0000 |
| 19 | CM32 | 0.0000 | 0.0221 | 0.1036 |
| 20 | CM33 | 0.0000 | 0.0010 | 0.0000 |

Table S6. Effect of the sequencing depth on mining SSRs

| Sequencing  depth | Number of SSRs | | | | | | | | | Total No. of polymorphic SSRs | Average Na |
| --- | --- | --- | --- | --- | --- | --- | --- | --- | --- | --- | --- |
|  | Na  2 | Na  3 | Na  4 | Na  5 | Na  6 | Na  7 | Na  8 | Na  9 | Na  10 |  |  |
| 2.5 | 36 | 8 | 4 | 1 | 1 | 0 | 0 | 0 | 0 | 50 | 2.460 |
| 5 | 293 | 126 | 41 | 14 | 2 | 0 | 0 | 0 | 0 | 476 | 2.542 |
| 7.5 | 665 | 254 | 110 | 31 | 12 | 1 | 1 | 0 | 0 | 1074 | 2.583 |
| 10 | 939 | 367 | 150 | 64 | 14 | 5 | 0 | 0 | 0 | 1539 | 2.611 |
| 12.5 | 1097 | 437 | 183 | 73 | 30 | 3 | 1 | 1 | 0 | 1825 | 2.641 |
| 15 | 1169 | 448 | 208 | 90 | 32 | 8 | 4 | 0 | 1 | 1960 | 2.681 |
| 17.5 | 1217 | 500 | 217 | 84 | 36 | 5 | 2 | 2 | 0 | 2063 | 2.669 |
| 20 | 1191 | 461 | 229 | 97 | 33 | 5 | 5 | 0 | 1 | 2022 | 2.695 |

Na: the number of alleles
